# Conservative Treatment of an Unusual Presentation of Iliopsoas Phlegmon Related to Infected Intrauterine Contraceptive Device

**DOI:** 10.1155/2024/9916070

**Published:** 2024-02-06

**Authors:** Giuleta Jamsari, Joseph Do Woong Choi, Benedict Kakala, Hillary Hu, Gideon Sandler

**Affiliations:** ^1^Department of Surgery, Westmead Hospital, Corner of Hawkesbury and Darcy Roads, Westmead, NSW 2145, Australia; ^2^Discipline of Surgery, Faculty of Medicine and Health, University of Sydney, Sydney, NSW 2050, Australia; ^3^Department of Obstetrics and Gynaecology, Westmead Hospital, Corner of Hawkesbury and Darcy Roads, Westmead, NSW 2145, Australia; ^4^Westmead Applied Research Centre, Faculty of Medicine and Health, University of Sydney, Westmead Hospital, Westmead, NSW 2145, Australia

## Abstract

Iliopsoas phlegmon/abscess is uncommon, and individuals often present with nonspecific symptoms. Diagnosis is often delayed and almost always requires advanced imaging techniques such as computed tomography or magnetic resonance imaging. We report a case of a 51-year-old woman who presented with right lower limb swelling and associated rash with imaging demonstrating iliopsoas abscess secondary to an infected intrauterine contraceptive device. This rare case highlights the nonspecific presentation of iliopsoas abscess and the need to consider unusual sources of infection such as an intrauterine contraceptive device in women presenting with iliopsoas phlegmon and abscess.

## 1. Introduction

Iliopsoas phlegmon/abscess is uncommon, with reported worldwide incidence of 12 new cases per year [1]. It is known to occur as result of hematogenous or lymphatic spread from a distant site, or it may be related to contiguous spread of nearby infectious process [2]. Individuals with iliopsoas phlegmon/abscess typically present with nonspecific symptoms, and due to the rarity of the disease, diagnosis is often delayed. The presentation can range from a mild infection with iliopsoas phlegmon to septic shock secondary to an iliopsoas abscess and its underlying cause. Advanced imaging techniques such as computed tomography (CT) are almost always required to diagnose an iliopsoas abscess [2].

We present a rare case of iliopsoas phlegmon secondary to infected intrauterine contraceptive device (IUCD). We highlight the nonspecific nature of presentation of iliopsoas inflammation and the need to consider various possible source(s) for iliopsoas abscess.

## 2. Case Report

A 51-year-old woman presented to the Emergency Department with a 3-week history of right lower limb swelling associated with pain and a rash extending from the right thigh to the right groin. She had seen her family doctor, who ruled out a lower limb deep vein thrombosis with a venous duplex Doppler ultrasound. She reported two weeks of intermittent night sweats and a 10 kg weight in the preceding six weeks. She denied abdominal or back pain, vaginal discharge, or dysuria. She had two previous lower segment caesarean sections and then had a copper IUCD inserted which had not been changed or removed for 15 years. She had been recently diagnosed with type 2 diabetes controlled with metformin. She denied recent overseas travel.

On physical examination, she appeared unwell. Her body mass index (BMI) was 39.8. She was tachycardic (119 bpm) and febrile (38.4°C), with a blood pressure of 177/92. Examination demonstrated a purpuric dermatosis extending from her right thigh to the groin and abdomen with associated pitting oedema. There were no clinically palpable masses. Her abdomen was soft with no associated tenderness, and there was no regional lymphadenopathy. A vaginal examination revealed foul smelling discharge with a copper IUCD in situ. The bimanual examination was unremarkable with no adnexal tenderness or palpable masses. The IUCD was removed at this time.

Biochemistry showed an elevated white cell count to 13.7 × 10^9^/L (3.9-11.1 × 10^9^/L) with neutrophils of 10.4 × 10^9^/L (2.0-8.0 × 10^9^/L) and C-reactive protein of 191 mg/L (<4 mg/L) consistent with an active inflammation. Vaginal and IUCD culture were positive for coliform species as well as heavy growth of mixed anaerobes on IUCD culture. Urine culture demonstrated *Escherichia coli*. Blood culture, syphilis, hepatitis, and human immunodeficiency screens were negative. Her serum carcinoembryonic antigen (CEA), cancer antigen 125, and CA 19-9 were normal.

**Figure 1:**
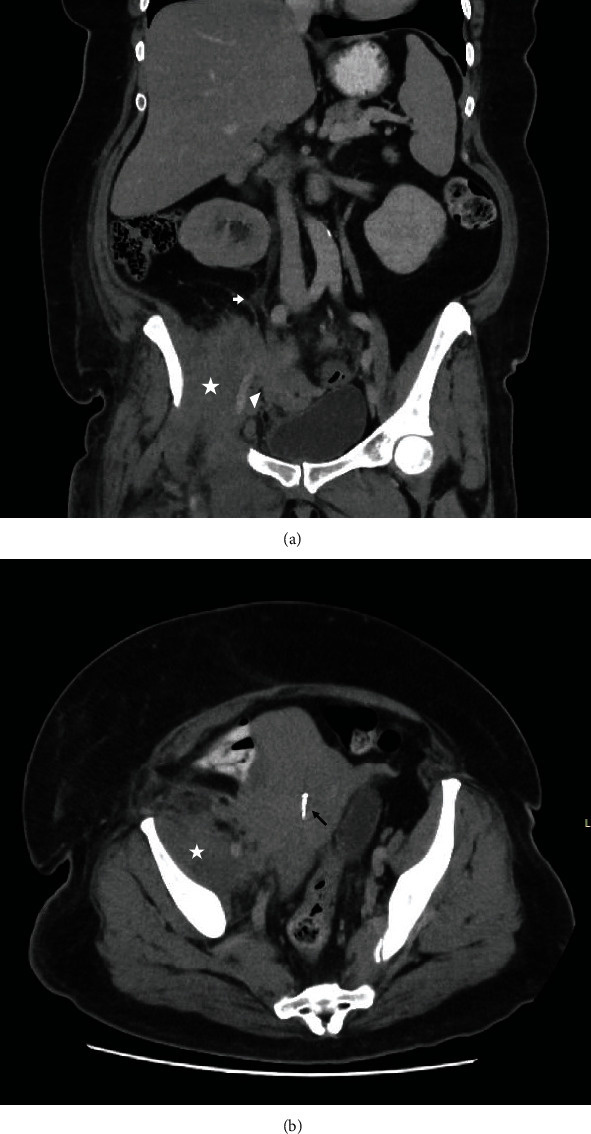
CT (a) coronal and (b) axial of the pelvis, demonstrating a 6 cm phlegmon in the right iliopsoas muscle (marked by star), with extrinsic compression of the right ureter (marked by white arrow), and the right external iliac vein (marked by triangle) and an intrauterine contraceptive device in situ (marked by black arrow).

**Figure 2:**
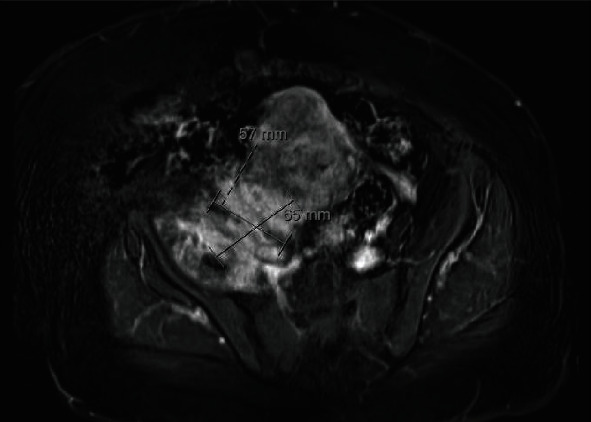
MRI of pelvis demonstrating a 6.5 × 5.7 cm ill-defined solid mass involving right iliopsoas muscle and extending to right uterus.

An ultrasound pelvis was limited due to body habitus but demonstrated uterine fibroids without ovarian or adnexal pathology. CT demonstrated a heterogenous thickening of right iliopsoas and iliacus muscles with moderate right pelvic sidewall oedema and fat stranding without a collection (Figure 1(a)). There was an associated irregular appearance of the uterus and right adnexa with oedema and stranding. There was mass effect with compression on the external iliac vein without thrombosis and the right ureter causing right-sided hydronephrosis (Figure 1(b)). These findings were likely diagnostic of an iliopsoas phlegmon. However, due to concerns of malignancy, these findings were corroborated by magnetic resonance imaging (MRI) pelvis which revealed a 6.5 cm ill-defined solid mass with no gas formation in the right pelvis involving the right edge of the uterus and extending laterally to the right iliopsoas muscle (Figure 2).

She was initially managed with five days of intravenous cefazolin (2 g three times a day) and then four days of intravenous ceftriaxone (2 g daily) under the guidance of the infectious disease team. The hydronephrosis did not require a ureteric stent due to normal renal function, and the external iliac vein compression was managed with subcutaneous therapeutic enoxaparin (100 mg twice a day). Given the imaging characteristics of the mass including a potential underlying malignancy, a CT-guided biopsy of the right iliopsoas mass was performed on day 9 of admission. There was no fluid aspirated. Histopathology demonstrated myofibroblastic reaction and patchy foci of mixed acute and chronic inflammation without malignancy, consistent with an inflammatory process. The bacterial, fungal, and mycobacterial cultures were negative. Her sepsis, lower limb swelling, and dermatosis subsequently improved, and she was discharged uneventfully after ten days of treatment with intravenous antibiotics.

## 3. Discussion

Iliopsoas abscesses are classified as primary or secondary. Primary iliopsoas abscess occurs with hematogenous or lymphatic spread from a distant or occult site, while secondary iliopsoas abscess occurs as result of direct extension from adjacent retroperitoneal or intra-abdominal organs [2, 3]. Secondary iliopsoas abscesses account for majority of cases and most commonly arise from intra-abdominal sources such as those with associated with Crohn's disease, appendicitis, or diverticulitis. It can also occur in association with musculoskeletal infection such as septic arthritis [2].

This case highlights a iliopsoas phlegmon/abscess secondary to an infected IUCD. Iliopsoas infection secondary to IUCD-related pelvic infection(s) is rare with only a few reported case reports. This was first described in 1993 in which a *Staphylococcus* psoas abscess occurred following a septic abortion with an IUCD in situ [4]. A subsequent case report describes an iliopsoas abscess due to *actinomycosis* secondary to an infected IUCD. Women who had undergone recent intrauterine instrumentation and use of copper containing IUCD and had an IUCD in situ for longer than 4 years had an increased risk of IUCD-related iliopsoas abscess [5, 6]. Our patient had a copper IUCD in situ for 15 years. IUCD-induced mucosal damage is thought to lead to bacterial translocation from the colonized IUCD which in-turn leads to psoas abscess formation [6].

In 1881, Mynter originally described the classic triad of back pain, limp, and fever in patients with iliopsoas abscess [7]. However, these patients often present with nonspecific and wide-ranging symptoms. This is reflected in our patient who presented with three weeks of right lower limb swelling and rash, making early diagnosis challenging. Cross-sectional imaging with CT and/or MRI remains the optimal method to diagnose and guide management of an iliopsoas abscess.

It appears that most of the reported cases of iliopsoas abscesses were managed with percutaneous or operative intervention. Two of the seven currently available case reports on iliopsoas abscess secondary to IUCD were managed with empirical antibiotics and percutaneous drainage [4–6, 8–10]. Stutz and Wilkinson reported a 38-year-old woman who had a psoas abscess related to a copper IUCD that had been present for over 10 years. This was associated with hydronephrosis requiring a nephrostomy. It was successfully drained under CT guidance, with cultures growing *Actinomyces* sp. and *Prevotella bivia* [6]. All other reported cases were managed with operative intervention with Cabot et al. and Scheepers et al. reported treatment with incision and drainage via extraperitoneal approach for IUCD-related psoas abscess [4, 10]. In another case, surgical approach with laparotomy, drainage of abscess, oophorectomy, and ileocolic resection were performed for a case of pelvic actinomycosis from a copper IUCD inserted 20 years prior to presentation [8].

In our patient, we report successful nonoperative treatment of iliopsoas phlegmon, with early recognition and removal of infected IUCD, intravenous antibiotics, and exclusion of malignancy with a biopsy. To date, this is the only case report that has reported successful management of iliopsoas phlegmon/abscess with conservative management.

## 4. Conclusion

Our case illustrates that secondary iliopsoas phlegmon/abscess can occur from contiguous spread from an IUCD-related pelvic infection and adds to the limited body of literature in diagnosing and managing this condition (this is the eighth reported case). It also highlights the feasibility of conservative management in IUCD-related iliopsoas abscess/phlegmon. Given the increased rate of IUCD insertions over time [11], this rare complication is expected to become more common. As such, when presented with iliopsoas abscess in female patient, clinicians should consider IUCD as possible source of infection.
